# Emerging Trends in Complications Associated with SARS-CoV-2 Infection

**DOI:** 10.3390/biomedicines12010004

**Published:** 2023-12-19

**Authors:** Elena Cecilia Rosca, Amalia Cornea, Mihaela Simu

**Affiliations:** 1 Department of Neurology, Victor Babes University of Medicine and Pharmacy Timisoara, Eftimie Murgu Sq. no. 2, 300041 Timisoara, Romania; amalia.cornea@yahoo.com (A.C.); mihaelasimu6713@gmail.com (M.S.); 2 Department of Neurology, Clinical Emergency County Hospital Timisoara, Bd. Iosif Bulbuca no. 10, 300736 Timisoara, Romania

The coronavirus disease 2019 (COVID-19) pandemic has presented a remarkable challenge to global health, sparking a surge in research aimed at understanding the multifaceted impacts of the virus. COVID-19 is a severe respiratory disease caused by severe acute respiratory syndrome coronavirus 2 (SARS-CoV-2). Despite the development of various vaccines and global programs, some patients may experience life-threatening complications. The acute complications can range from respiratory failure to cardiac and cardiovascular injury, liver and renal disorders, neurologic manifestations, secondary infections, coinfections, and disseminated intravascular coagulation. However, COVID-19 is not only a short-term infection, but can also present long-term complications. Post-COVID-19 conditions are more common after severe illness, but any patient can experience long COVID, even those with mild or no symptoms.

Recent studies have delved into diverse aspects of the impact of the virus, shedding light on its metabolic, cardiovascular, pediatric, and neurological dimensions. 

Acute respiratory distress syndrome (ARDS) is more common than initially thought, with 10% of intensive care units patients and 23% of mechanically ventilated patients meeting the criteria [1]. Hospital mortality rates range from 35 to 45%, with a 31% mortality rate even in rapidly resolved cases [2]. The COVID-19 pandemic has highlighted this critical care condition [3]. Among ARDS predictors in hospitalized COVID-19 patients, body mass index (BMI), body fat percentage (BF%), and visceral fat (VF) are significant characteristics, with obesity emerging as a crucial risk factor for ARDS. Additionally, admission predictors of ARDS have been delineated: having a very high BMI, SaO_2_ < 87.5, IL-6 > 59.75, a low lymphocyte count, being of female sex, and aged <68.5. Obesity was found to be a significant risk factor for ARDS clinical deterioration. These findings support the need for targeted interventions in obese patients to mitigate the risk of ARDS [4]. 

Prognostic markers also include the evaluation of N-terminal pro-brain natriuretic peptide (NT-pro-BNP) [5]. Studies have revealed that NT-pro-BNP levels and other clinical parameters are independently associated with in-hospital mortality [6,7]. Furthermore, mortality has been reported to be increased in patients of older age, with a lower GCS and PaO_2_/FiO_2_ ratio, elevated D-dimer values, INR, creatinine values, and shorter PT. They showed an increased frequency of heart failure and higher NT-pro-BNP values. Multivariable logistic regression analysis demonstrated that higher NT-pro-BNP values and lower PT and PaO_2_/FiO_2_ at admission were independent predictors of mortality during hospitalization. Although further longitudinal studies are needed in order to confirm these findings, the authors underscore the significance of monitoring NT-pro-BNP levels in COVID-19 pneumonia patients to identify those at highest risk [7]. 

A common complication in COVID-19 patients is acute kidney injury (AKI) [8], with older age, higher admission serum creatinine, an elevated Sequential Organ Failure Assessment (SOFA) score, elevated D-dimer, elevated C-reactive protein (CRP) on day 2, mechanical ventilation, vasopressor requirement, and azithromycin usage being significant risk factors [9]. However, the combined use of Tocilizumab and corticosteroids has been independently associated with reduced AKI risk. Therefore, the early administration of anti-inflammatory agents may improve clinical outcomes, and anti-inflammatory agents should be considered when managing COVID-19-associated AKI. Nonetheless, the association of AKI with the usage of common anti-COVID-19 drugs (e.g., remdesivir, tocilizumab, and lopinavir/ritonavir) should be further investigated [10], and further research is required to assess the risks and benefits of Tocilizumab treatment in critically ill COVID-19 patients with renal impairment [11].

The COVID-19 pandemic has highlighted high-risk groups for poor COVID-19 outcomes [12], including elderly patients receiving hemodialysis, with factors such as age, immunosenescence, and increased exposure to infection sources influencing COVID-19 incidence, severity, and mortality. Preventative measures such as regular screening and vaccination programs have been reported to be crucial to reducing COVID-19 cases and complications, especially for elderly hemodialysis patients. The pandemic has led to the development of medications for treating viral and inflammatory diseases, but elderly hemodialysis patients have been underrepresented in many trials. Nonetheless, contemporary treatments for COVID-19 in this population, with an up-to-date guide, are now available [13]. 

Shifting the focus to another critical aspect, the elevated serum urea-to-creatinine ratio (UCR) emerges as a potential proxy for hypovolemia, with a 5-unit increase linked to an 8% rise in all-cause mortality. Notably, patients with a baseline UCR > 40 face higher odds of in-hospital death when experiencing > 10 mmol/L increases in serum sodium within the first week [14]. These findings underscore the importance of monitoring sodium levels and implementing targeted interventions for at-risk patients [15].

The COVID-19 pandemic has led to various levels of prevalence and geographical areas of ARDS in hospitalized patients, with unclear optimal respiratory support and high mortality rates due to early intubation experiences and the lack of international guidelines for noninvasive modalities. Numerous studies have attempted to compare supplemental high-flow nasal cannula (HFNC) oxygen and normal oxygen therapy (NIV) in COVID-19 patients, with inconclusive results [16,17]. However, other researchers have evaluated the use and success rate of helmet continuous positive airway pressure (hCPAP) in regular medical wards, offering a tailored approach for COVID-19-associated ARDS, with a computed hCPAP-f Score emerging as a valuable tool for predicting hCPAP failure, offering a nuanced approach to patient management. Factors associated with hCPAP use include a PaO_2_/FiO_2_ ratio < 270, IL-6 serum levels over 46 pg/mL, AST > 33 U/L, LDH > 570 U/L, age > 78 years, and neuropsychiatric conditions. The failure of hCPAP was associated with being of male sex, polypharmacotherapy, a platelet count < 180 × 109/L, and a PaO2/FiO2 ratio < 240 [18].

The clinical presentation of SARS-CoV-2 varies [19], with respiratory tract infections and multi-organ damage being common findings. Proper investigation is crucial due to the high risk of systemic inflammation, such as myositis and myocarditis, which can lead to fatal outcomes. Among the atypical presentations of COVID-19, myocarditis, mimicking autoimmune disease, necessitates further exploration into the autoimmune aspects of COVID-19 [20].

Although parosmia was a previously known disorder with limited clinical interventions, it is only since the COVID-19 epidemic that researchers have had to deal with an ever-increasing number of patients [21], contributing to an expansion of knowledge and innovative techniques [22]. For example, researchers have confirmed the efficacy of combining ultramicronized palmitoylethanolamide and Luteolin (umPEA-LUT) with olfactory training as a therapy for COVID-19-related quantitative smell alteration, emphasizing the importance of addressing both the central and peripheral aspects of olfactory dysfunction [23].

Recently, at least 74 different clinical manifestations were demonstrated to be associated with COVID-19 [19]. Parsonage–Turner syndrome (PTS), an inflammatory disorder of the brachial plexus with immune-mediated causes, was also reported following SARS-CoV-2 infection. A systematic review revealed 26 cases of PTS in patients who had previously contracted COVID-19 [24]. The spectrum was heterogeneous, with 93.8% experiencing severe pain, 80.8% suffering motor deficit, and 53.8% undergoing muscle wasting. Paresthesia was noted in 46.2% of PTS individuals, and sensory loss in 34.6%. This review emphasizes the need for a high index of suspicion for PTS and underscores the necessity for standardized investigation and reporting protocols [24].

SARS-CoV-2 does not show selective tropism to the respiratory system; it has widespread effects on many tissues, including endocrine gland impairment [25,26]. Recent research has examined the activity of the thyroid and adrenal glands through 18F-FDG PET/CT scans, revealing a persistent low 18F-FDG uptake in adrenal glands, suggesting chronic hypofunction. Conversely, the thyroid’s metabolic activity, initially comparable to normal subjects, exhibited an intriguing shift post recovery, hinting at the onset of inflammatory thyroiditis. These observations advocate for heightened vigilance regarding the pituitary–adrenal axis and thyroid functionality in COVID-19 patients during infection and recovery [27]. 

Regarding pediatric cases, researchers have also investigated the prevalence of respiratory viruses and their coinfections in children with severe respiratory infections (SARIs) [28]. The five most frequent coinfections identified were human rhinovirus (hRV)/SARS-CoV-2 (17.91%), hRV/respiratory syncytial virus (RSV) (14.18%), RSV/SARS-CoV-2 (12.69%), hRV/human bocavirus (BoV) (10.45%), and hRV/adenovirus (AdV) (8.21%) [29]. These findings offer valuable insights into the complexity of pediatric respiratory infections and the need for tailored interventions.

Bibliometric analysis is an approach that uses a set of quantitative methods to measure, track, and analyze the scholarly literature [30], providing an overview of a field of research. It has been used in SARS-CoV-2-infection-related research. For example, a bibliometric review exploring studies that assessed SARS-CoV-2 antibody responses in the pediatric population highlighted collaborative networks, international interactions, and key findings regarding antibody titers and correlations with immune markers. This study offers insights into future research directions in pediatric SARS-CoV-2 antibody responses [31]. 

Together, these papers provide a wealth of information, revealing the many facets of COVID-19’s effects on various physiological systems. Each study adds a piece to the puzzle, including topics such as neurological symptoms, pediatric responses, metabolic abnormalities, and cardiovascular and metabolic consequences. As we examine these findings, the importance of standardized approaches, vigilant monitoring, and tailored interventions becomes clear. The multidisciplinary nature of these studies underscores the need for collaborative efforts and for pushing the boundaries of knowledge to better understand not only the SARS-CoV-2 infection but also any new pathogens.
